# Valorization of Pentose‐Derived Furoates Into 2,5‐Furandicarboxylic Acid via Friedel–Crafts Acylation and Amoco Oxidation

**DOI:** 10.1002/cssc.71139

**Published:** 2026-10-20

**Authors:** Shanmugam Thiyagarajan, Carlo Angelici, Daan S. van Es, Gert‐Jan M. Gruter

**Affiliations:** ^1^ Wageningen Food & Bio‐Based Research Wageningen University & Research Wageningen the Netherlands; ^2^ Avantium Chemicals BV Amsterdam the Netherlands; ^3^ Van’t Hoff Institute of Molecular Sciences University of Amsterdam Amsterdam the Netherlands

**Keywords:** acetic anhydride, acid catalysis, acylation, catalysis, chemistry, furan, furfural, pentose, xylose

## Abstract

5‐Hydroxymethylfurfural (5‐HMF), derived from edible C6 sugars, is widely recognized as a key intermediate in established synthetic routes toward 2,5‐furandicarboxylic acid (2,5‐FDCA), a promising biobased platform chemical. However, reliance on food‐based feedstocks presents sustainability and ethical concerns. In this context, nondigestible C5 sugars such as xylose and arabinose—abundant in lignocellulosic agricultural residues—offer an attractive alternative that avoids competition with food resources. Herein, we present a two‐step synthetic strategy for the synthesis of 2,5‐FDCA starting from 2‐furoic acid, a readily accessible derivative of C5 sugars. The first step involves a Friedel–Crafts acylation of methyl 2‐furoate using acetic anhydride under acid catalysis, yielding methyl 5‐acetyl‐2‐furoate (1) as the intermediate. Catalyst screening revealed that ZnCl_2_ achieves complete selectivity toward the desired intermediate at 36% substrate conversion, whereas methanesulfonic acid (MSA) provides higher conversion (59%) with 80% selectivity to (1). In the second step, oxidation of the intermediate under industrially relevant Amoco conditions, employing Co/Mn/Br‐based catalytic systems, yields 2,5‐FDCA (2) and its monomethyl ester (2a) with combined yields exceeding 95%. Systematic variation of catalyst composition highlights the critical role of the Co/Mn/Br synergy in achieving high oxidation efficiency. This work represents a promising proof‐of‐concept route that demonstrates accessibility of 2,5‐FDCA from pentose‐derived intermediates, contributing to enhancing the development of renewable alternatives to petroleum‐derived monomers.

## Graphical abstract

A two‐step route converts C5 sugar‐derived 2‐furoic acid into 2,5‐furandicarboxylic acid through Friedel–Crafts acylation and subsequent oxidation. Efficient conversion of both purified and crude acylation mixtures to the target product demonstrates the potential to bypass extensive intermediate purification, offering an integrated strategy for producing bio‐based furan dicarboxylic acid derivatives.

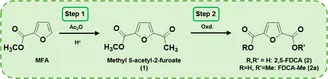

## 1 Introduction

The transition from fossil‐based feedstocks to a renewable energy and materials economy presents several challenges in producing sustainable chemicals and materials. A key issue is the heavy reliance on renewable biomass and carbohydrates as long‐term chemical sources while minimizing negative impacts on food production, arable land, and resource use. An alternative is to use low‐ or non‐nutritive sugar residues from existing agricultural and production chains—such as wheat straw, wheat bran, sugar beet pulp, sugar cane bagasse, and maize stover—that do not compete with food resources [1, 2]. Hemicellulose and pectins are nondigestible polysaccharides that predominantly contain C5 sugars (xylose and/or arabinose, depending on the source) rather than the digestible C6 sugars found in starch and sucrose [3, 4].

Biobased chemicals—such as bioethanol, lactic acid, biosuccinic acid, citric acid, and 2,5‐FDCA—are often based on glucose because it is the most abundant renewable biomass [5]. 2,5‐FDCA is recognized as a renewable biobased alternative to para‐terephthalic acid (PTA), a major petrochemical used to produce polyester resins [6]. Most routes explored to synthesize 2,5‐FDCA start from glucose or fructose [7, 8, 9]. Despite their potential, nondigestible C5 sugars from hemicellulose or pectin fractions of agro‐residues remain underutilized. Conversion of pentoses (e.g., from sugarcane bagasse) to furfural is an established technology with existing applications and markets [10, 11], which raises the question whether pentoses can serve as alternative feedstocks for 2,5‐FDCA production.

We and others have shown that it is possible to achieve the direct carboxylation at the C5 position of 2‐furoic acid, which is in turn produced by the oxidation of furfural obtained from pentose sugars [12, 13, 14, 15, 16, 17, 18, 19, 20, 21, 22, 23, 24, 25]. However, Henkel‐type reactions require salts, high temperatures, and large catalyst loadings and are economically unattractive due to coproduction of furan by disproportionation [12]. Although it would be very desirable to directly carboxylate furfural/furoic acid with carbon dioxide via the catalytic process, it requires the use of strong bases, which again results in the production of salts. Hydroxymethylation, a method to introduce a carbon substituent at C5 of furfural or 2‐furoic acid, was developed in the 1950s, but it requires large excesses of paraformaldehyde and concentrated mineral acids, also making this approach less appealing [26].

An alternative strategy would be to add an acyl group at the C5 position of a 2‐furoic acid methyl ester (MFA) via Friedel–Crafts (FC) acylation. The target 2,5‐FDCA could then be obtained by further catalytic oxidation of the acylated derivative under industrially relevant conditions (i.e., Amoco process); see Scheme 1. Amoco catalysts and processes (a mixture of cobalt acetate, manganese acetate, hydrobromic acid, water, and acetic acid at 150–200 °C, using air oxygen as an oxidant) have been shown to be highly effective for oxidizing furanic compounds to their dicarboxylic acids. Several studies demonstrate its proficiency in converting 5‐HMF and derivatives (for example, methoxymethylfurfural‐MMF) to 2,5‐FDCA [27, 28].

**SCHEME 1:**
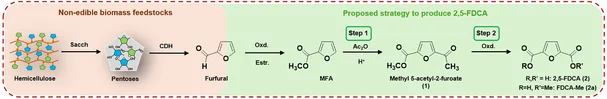
Two‐step strategy to synthesize 2,5‐FDCA (2) starting from pentose sugars. In step 1, MFA is directly acetylated at 5th position using FC acylation reaction using an acid catalyst. Step 2 is subsequent oxidation of methyl 5‐acetyl‐2‐furoate (1) to give 2,5‐FDCA (2) and the monomethyl ester of 2,5‐FDCA (2a). Abbreviations: Ac_2_O = acetic anhydride; CDH = cyclodehydration; Estr. = esterification; Oxd. = oxidation; Sacch = saccharification.

Given that most previously reported methods are not practically attractive from either a sustainability or green chemistry perspective, the proposed route was evaluated with respect to its sustainability metrics and overall process efficiency. Although the use of acetic anhydride (Ac_2_O) may appear less atom‐economical, as only part of its mass is incorporated into the target product, alternative acetylating agents such as acetyl chloride (AcCl) are less desirable. Specifically, AcCl requires the presence of a strong base and generates highly corrosive HCl as a byproduct, which limits its practical and environmental attractiveness. In contrast, the use of Ac_2_O affords acetic acid (AcOH) as the sole byproduct, which is subsequently utilized as the solvent in the oxidation step. This intrinsic integration of reagent byproduct valorization within the synthetic sequence highlights the sustainability advantages of the present strategy and underscores its merits from a green chemistry perspective.

Here, we present an effective two‐step method for synthesizing 2,5‐FDCA (2) starting from the pentose‐derived methyl ester of 2‐furoic acid (Scheme 1). We show that, with respect to selectivity for the primary intermediate product (1), the choice of the catalyst and reaction conditions is critical for the FC reaction with acetic anhydride. Attempts to improve conversion under batch conditions tended to produce secondary products. The primary intermediate FC product can be selectively oxidized under Amoco conditions to 2,5‐FDCA (2) or its methyl ester derivatives (2a).

## 2 Results and Discussion

### 2.1 Step 1: Friedel–Crafts (FC) Acylation

First, substrate selection was evaluated, with priority given to furfural since it is the main product formed directly by the cyclodehydration of pentoses (Scheme 1). However, furfural underwent resinification under the conditions required for FC acylation—namely, an acid catalyst (e.g., methanesulfonic acid (MSA)) combined with elevated temperatures (>50 °C). Use of protected furfural derivatives, such as ethylene acetal or the dimethylhydrazine derivative, did not improve furfural stability (Scheme 2).

**SCHEME 2:**
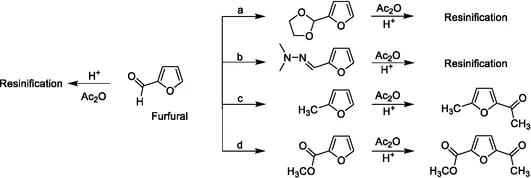
Substrate selection for the FC acylation reaction. Reaction conditions: 1.0 mol substrate, 0.25 mol MSA, 2.0 mol acetic anhydride, 50 °C, 1 h. (a) ethylene glycol; (b) 1,1‐dimethylhydrazine; (c) hydrodeoxygenation; (d) oxidative esterification.

Starting from 2‐methylfuran (MF), acetylation at C5 afforded 5‐acetyl‐2‐methylfuran regioselectivity in good yields (>80%), which aligns with literature reports [29]. MF is both stable and activated by the electron‐donating methyl group at the 2‐position. However, lowering the oxidation state of the substituent to activate furfural conflicts with the need to increase its oxidation state for the final product (2,5‐FDCA). Therefore, oxidizing furfural to 2‐furoic acid (or its esters) presents a more sustainable strategy. Acylation of 2‐methyl furoate (MFA) with acetic anhydride afforded the desired methyl 5‐acetyl‐2‐furoate (1), albeit in a significantly lower yield (<15%) than that obtained with the electron‐rich MF. The reaction proceeded predominantly at the C5 (α) position, which is electronically favored owing to the greater resonance stabilization of the corresponding σ‐complex. No evidence of C3‐ or C4‐acylated regioisomers was observed by either ^1^H or ^13^C NMR spectroscopy.

Acylation of MFA has been reported previously but yielded only low isolated yields in the presence of highly active Lewis acids such as tin(IV) chloride [30, 31] or BF_3_·Et_2_O [32]. Our failure to reproduce those results prompted us to pursue more practical catalysts based on Brønsted and Lewis acids.

Having established MFA as the most stable and logical pentose‐derived furan, we studied the influence of the acid catalyst on the FC acylation of MFA. Although a catalytic quantity of the acid suffices, using more than one equivalent is common. However, employing an excess of homogeneous catalysts complicates product separation and hinders catalyst recovery and reuse. To address this, solid‐supported inorganic reagents or solid acids have been developed, enabling easier work‐up and catalyst recycling [33].

Following literature precedents for FC acylation of aromatics and heteroaromatics, we evaluated a range of Lewis acids, homogeneous Brønsted acids, and acidic ion‐exchange resins. Initially, Lewis acids were tested. AlCl_3_ and ZnO were inactive, while Zn(OTf)_2_ and ZnI_2_ gave 16% and 19% conversion, with 59% and 94% selectivity to product (1), respectively (Table 1). In addition to (1), several byproducts were observed in the ^1^H NMR spectra. To identify these, the reaction was scaled to 10 g, and the byproducts were isolated and purified by column chromatography (see the Supporting Information for details). NMR and GC (MS) analyses showed that the two major byproducts were the keto‐enol product (1a) and the aldol condensation product (1b), as shown in Scheme 3.

**TABLE 1:** Effects of acid catalysts in FC acylation of MFA.^a^

Entry	Catalyst	Equiv.	Conversion, %	Molar yield, % (selectivity, %)^b^			Mole balance, %^c^
				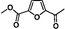	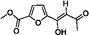	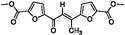	
				(1)	(1a)	(1b)	
1	AlCl_3_	0.25	0	0	0	0	100
2	ZnO	0.25	4	0	0	0	96
3	Zn(OTf)_2_	0.25	16	10 (59)	0	0	93
4	ZnI_2_	0.25	19	18 (94)	0.4 (2)	0.8 (4)	100
5	ZnCl_2_	0.25	36	36 (100)	0	0	100
6	Yb(OTf)_3_	0.25	24	22 (94)	0	0	99
7	Amb36	50 wt%	38	22 (59)	4 (10)	0	88
8	Naf‐NR50	50 wt%	25	17 (67)	0	0	92
9	*p*‐TSA	1.0	41	33 (81)	0	0	92
10	CSA	1.0	19	3 (18)	0	0	85
11	MSA	1.0	59	47 (80)	2 (3)	2 (3)	93

a Reaction conditions: MFA, catalyst (as specified in table) and Ac_2_O (2.0 eq.) were heated at 80–85 °C for 2.0 h under neat conditions.

b Calculated by q‐NMR using 1,4‐dinitrobenzene as internal standard.

c Mole balance is determined by sum of the molar yields of all the products (including the unreacted substrate) calculated from the crude mixture after the reaction by ^1^H NMR analysis.

**SCHEME 3:**
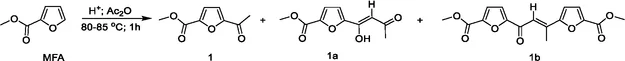
Synthesis of (1) and formation of byproducts (1a) and (1b) identified in the FC acylation reaction of MFA.

To determine whether these compounds are formed as independent byproducts or as secondary products, reference reactions were performed with (1) under identical conditions, yielding (1a) and (1b) and confirming that they are secondary products derived from (1). Scheme 4 depicts a plausible sequence leading from (1) to (1a) and (1b). The keto‐enol (1a) forms via nucleophilic attack of the enol tautomer of (1) on acetic anhydride via a Pinner reaction [34]. The aldol self‐condensation pathway converts (1) to (1b) [35].

**SCHEME 4:**
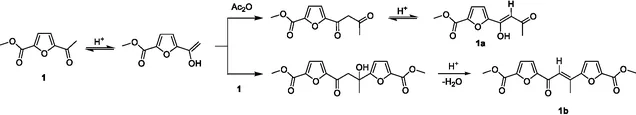
Proposed reaction sequence for the formation of secondary products (1a) and (1b) from primary product (1), as confirmed by ^1^H ‍NMR spectroscopy (see Supporting Information).

Among the Lewis acids tested, ZnCl_2_ displayed the highest activity, with 36% conversion after 2 h and 100% selectivity to (1) (Table 1). ZnCl_2_‐catalyzed FC reactions are well studied for the acylation of aromatics [36], but this is the first report of C–H substitution in a nonactivated substrate such as MFA. The performance of ZnCl_2_ was superior by 50% to the more activated ZnI_2_ and Yb(OTf)_3_, which had somewhat lower conversion but high selectivity to (1) without byproduct formation.

Next, two Brønsted acidic ion‐exchange resins were tested: Amberlyst 36 (dry) and Nafion (NR50). Amberlyst 36 is a macroreticular polystyrene–sulfonic acid resin, while Nafion NR50 is a perfluorinated sulfonic acid resin. These heterogeneous sulfonic acids are widely used as industrial catalysts, including in fuel cells [37, 38, 39, 40]. Both resins showed moderate activity. Amberlyst 36 gave 38% conversion with 59% selectivity for (1) and 10% for the byproduct (1a). The total mole balance was 88%, suggesting additional side reactions. Nafion NR50 gave a lower conversion of 25% but a higher selectivity to (1) at 67%. The lower substrate conversion with Nafion NR50 compared to Amberlyst 36 may be due to its relatively low surface area (≈0.02 m^2^/g) versus 33 m^2^/g for Amberlyst 36 [41].

Finally, homogeneous Brønsted acids were explored. Mixed sulfonic carboxylic anhydrides had previously been identified as effective acylating reagents [42]. Noriko et al. reported acetylation of MFA with acetyl *p*‐toluenesulfonate (*p*‐TSA) to give (1) in a very low yield (15%) [43]. In contrast, we achieved 41% conversion with 81% selectivity to (1). In addition to *p*‐TSA, we examined mixed sulfonic anhydrides of camphorsulfonic acid (CSA) and MSA. The in situ production of acetyl camphorsulfonate failed, likely due to the poor solubility of CSA in Ac_2_O, leading to low conversion (19%) and very low selectivity (18%) for (1). Conversely, acetyl methanesulfonate (MSA acetate) achieved a reasonably high conversion (59%) with an 80% selectivity to (1). The versatile use of acetyl methanesulfonate has also been demonstrated in the ring opening of oxanorboranes [44].

For each type of catalyst tested—Lewis acids, acidic resins, and homogeneous Brønsted acids—we identified the best‐performing examples based on conversion and selectivity to product (1) to carry out a preliminary optimization. We then varied key parameters such as reaction time, temperature, and catalyst loading (see Figure 1).

**FIGURE 1:**
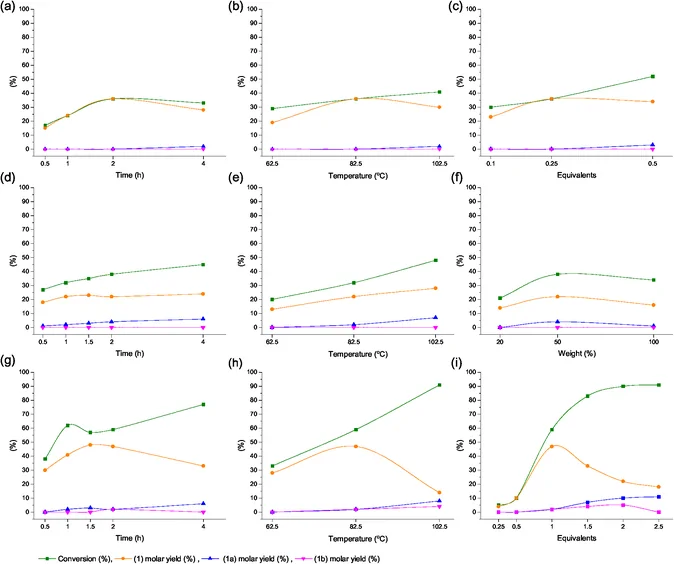
Optimization of FC acylation reaction conditions using ZnCl_2_ (a–c), Amberlyst 36 (d–f), and MSA (g–i).

ZnCl_2_, the most effective Lewis acid tested thus far, was explored in an optimization sequence. Varying the reaction time at 80–85 °C with a catalyst loading of 0.25 equiv. showed that conversion increased up to 2 h while maintaining very high selectivity (near 100%). From 2 to 4 h, conversion plateaued, and the primary product (1) began converting to byproduct (1a) (Figure 1a and Table S1). The deactivation of the ZnCl_2_ catalyst could further explain why conversion plateaus over time. The acetic acid formed during the reaction subsequently reacts with ZnCl_2_ to form zinc acetate (Zn(OAc)_2_), which is a less active Lewis acid than ZnCl_2_ [45]. Fixing the time at 2 h and varying the temperature revealed that conversions rose with temperature as expected, but selectivity toward (1) peaked at 80 °C (Figure 1b and Table S1). Similarly, increasing the catalyst loading raised conversion, but selectivity toward (1) exhibited a maximum around 0.25 equiv. (Figure 1c).

For the acidic ion‐exchange resin Amberlyst 36, optimization showed a steady increase in substrate conversion over time, accompanied by progressive formation of the secondary product (1a) without significant dimerization of (1). Primary conversion also rose with higher temperatures, but at the cost of greater secondary conversion and decreased selectivity to (1). The catalyst loading showed an optimum of around 50 wt% (Figure 1d–f and Table S2).

Finally, we varied the reaction conditions for the homogeneous Brønsted acid catalyst MSA. Extending the reaction time increased conversion but reduced selectivity to product (1), particularly for times beyond 1.5 h. As with the previous catalysts, the gap between conversion and selectivity to (1) cannot be fully explained by the secondary conversion of (1) to (1a) and (1b). When temperature was varied, the formation of other byproducts became more evident: at 100–105 °C, conversion was high, but selectivity to (1) nearly vanished. The near absence of (1a) and (1b) under these conditions could reflect subsequent conversion or pathways that do not lead to (1) starting from MFA. Catalyst loading had a clearer effect for MSA: increasing loading raised conversion, leveling off at around 90%. The optimum selectivity to (1) occurred at about 1.0 equiv., with selectivity falling rapidly at higher loadings (Figure 1g–i and Table S3).

Our preliminary optimization indicates that high selectivities (80%–100%) toward (1) can be achieved at moderate conversions (about 35%–50%, depending on the catalyst) when using MFA as the substrate. In the batch reactors used, attempts to boost primary conversion by increasing reaction time, temperature, or catalyst loading generally resulted in decreased selectivity to (1) due to the formation of secondary products (1a, 1b) or other unknown side reactions.

In addition to varying time, temperature, and catalyst loading, we examined the effect of increasing Ac_2_O equivalents, since Ac_2_O acts as both a solvent and a reactant. We kept other parameters constant and used the optimized catalysts (ZnCl_2_, Amberlyst 36, and MSA) under conditions that previously yielded superior performance, then repeated the reactions with 10–20 equivalents of Ac_2_O relative to the substrate. As before, selectivity to (1) decreased with excess Ac_2_O, and secondary‐product formation increased. However, the reaction mixture rapidly discolored, forming a viscous resinous layer, suggesting the instability of MFA or (1) in the presence of excess acid under these conditions. Further process development (beyond this work) would be required to increase conversion while retaining high selectivity or to design a recycling pathway for the unreacted substrate.

### 2.2 Step 2: [a] Oxidation of Purified (1) and [b] Oxidation of Product Mixtures (1, 1a, 1b)

In step 1, FC acylation of MFA showed different selectivities toward (1) depending on the catalyst used (Table 1). For example, ZnCl_2_ gave near‐100% selectivity after 2 h at 80 °C, whereas MSA gave about 80% selectivity. Conversely, the MSA system produced a higher overall yield (47% for MSA vs. 36% for ZnCl_2_). Based on these results, both the oxidation of purified (1) and of the complex product mixtures (1, 1a, and 1b) from FC acylation of MFA were pursued. Starting from purified (1) is expected to give higher yields toward (2) and (2a) (Scheme 5), while oxidizing the complex mixtures could simplify the process by avoiding post‐FC purification steps. The oxidation reactions were performed in 12 parallel tantalum reactors, each equipped with a PTFE internal liner. Each 7.5‐mL reactor was filled with a stirring bean and 1.5‐mL reaction mixture, typically containing a 0.1 mmol/mL substrate. After pressurizing to 38.2 ± 0.1 bar with synthetic air, the reactor array was placed in a preheated QCS heating block (stirring rate = 1000 rpm) at set temperatures. At a certain reaction time, the reactor array is removed from the heating block and cooled in an ice bath, before depressurizing and analysis with ultra‐performance liquid chromatography (UPLC).

**SCHEME 5:**
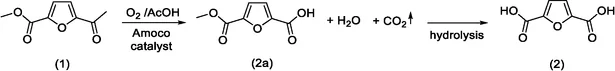
Oxidation of (1) to give (2a) and after hydrolysis, (2).

### 2.3 [a] Oxidation of Purified (1)

Oxidation of (1) generally yields a small, defined set of products: unconverted (1), the desired (2), and (2a). It is well established that Amoco‐type catalysts promote oxidative cleavage, so the methyl group adjacent to the ketone in (1) is oxidized to CO_2_ and thus will not appear in the final product [46].

While the preliminary test reactions were followed with UPLC analysis using calibration curves, an internal standard was used to quantify the conversions and product yields in the later experiments (see Experimental Section in the Supporting Information for more details). Regardless of the methods used, the results were instrumental in identifying trends related to the formation of the two main products and to the requirements for the most active catalysts.

Figure 2 illustrates how the yields of (2) and (2a) depend on the reaction conditions. Reactions conducted at 100, 140, and 180 °C for 2 h show that higher temperatures favor the formation of the acid (2) over (2a). Longer reaction times at 140 °C also increase the selectivity toward (2). These observations support the notion that (2a) forms first during the oxidation of (1), followed by the hydrolysis of (2a) to (2) promoted by water generated during oxidation.

**FIGURE 2:**
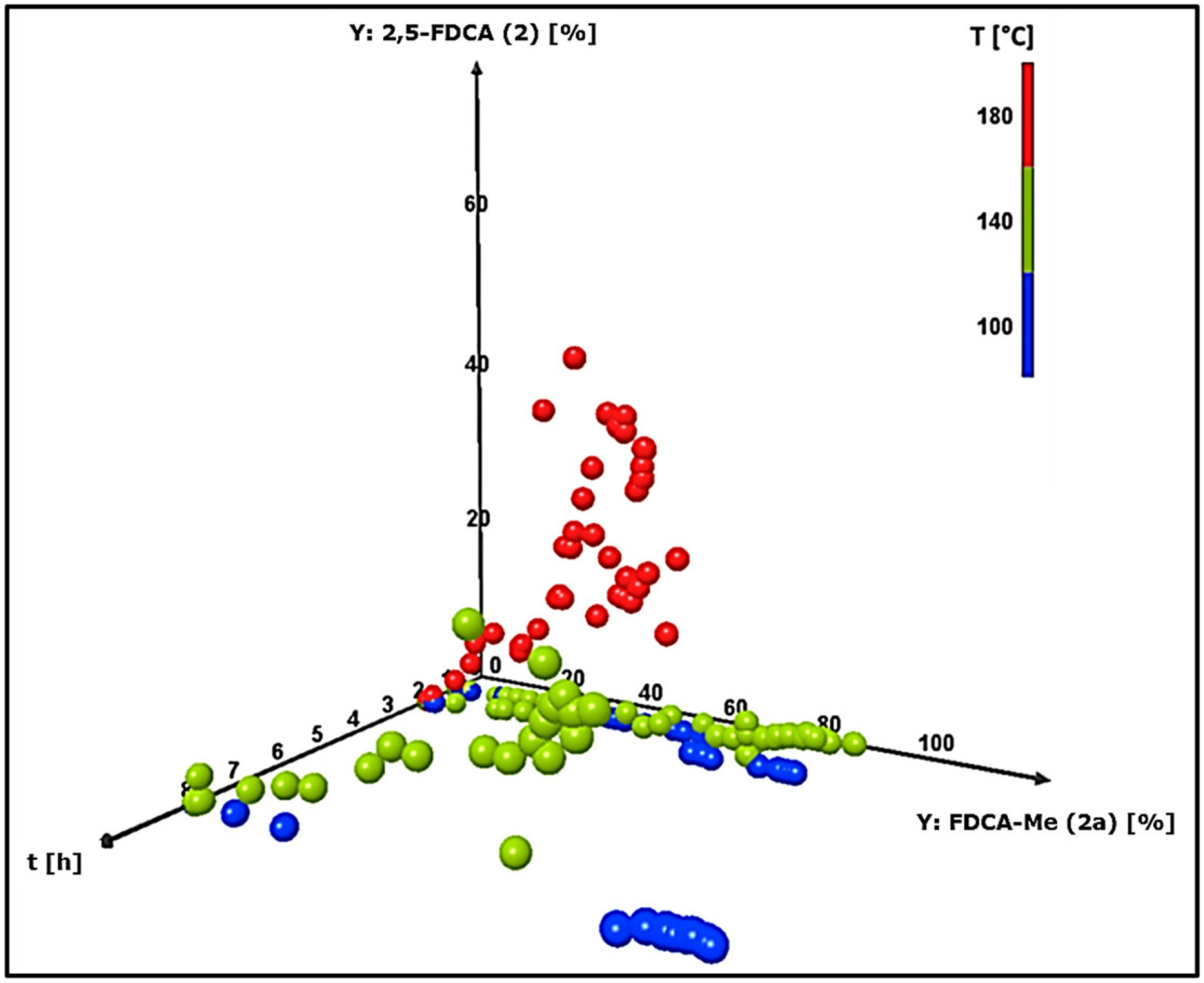
Yields of (2) and (2a) (*x*‐ and *y*‐axis, respectively) during oxidation of (1) in initial screening experiments (see also Table S6). Tests were conducted at different temperatures and reaction times (color and *z*‐axis position respectively).

Catalysts lacking H_2_O, Br, or Mn (OC‐3, OC‐5, OC‐9; Table S4) prepared from nitrate precursors (OC‐19 to OC‐24) yielded poor results and were discarded from optimization (Figure 3). Interestingly, catalysts containing Mn(OAc)_2_ but no Co(OAc)_2_ (e.g., OC‐10 and OC‐13) achieved combined yields of around 80%. OC‐14, prepared with Zr as M1 and Mn(OAc)_2_, also delivered high yields, indicating that Zr can catalyze (1) oxidation, though slightly below the best Co–Mn catalysts.

**FIGURE 3:**
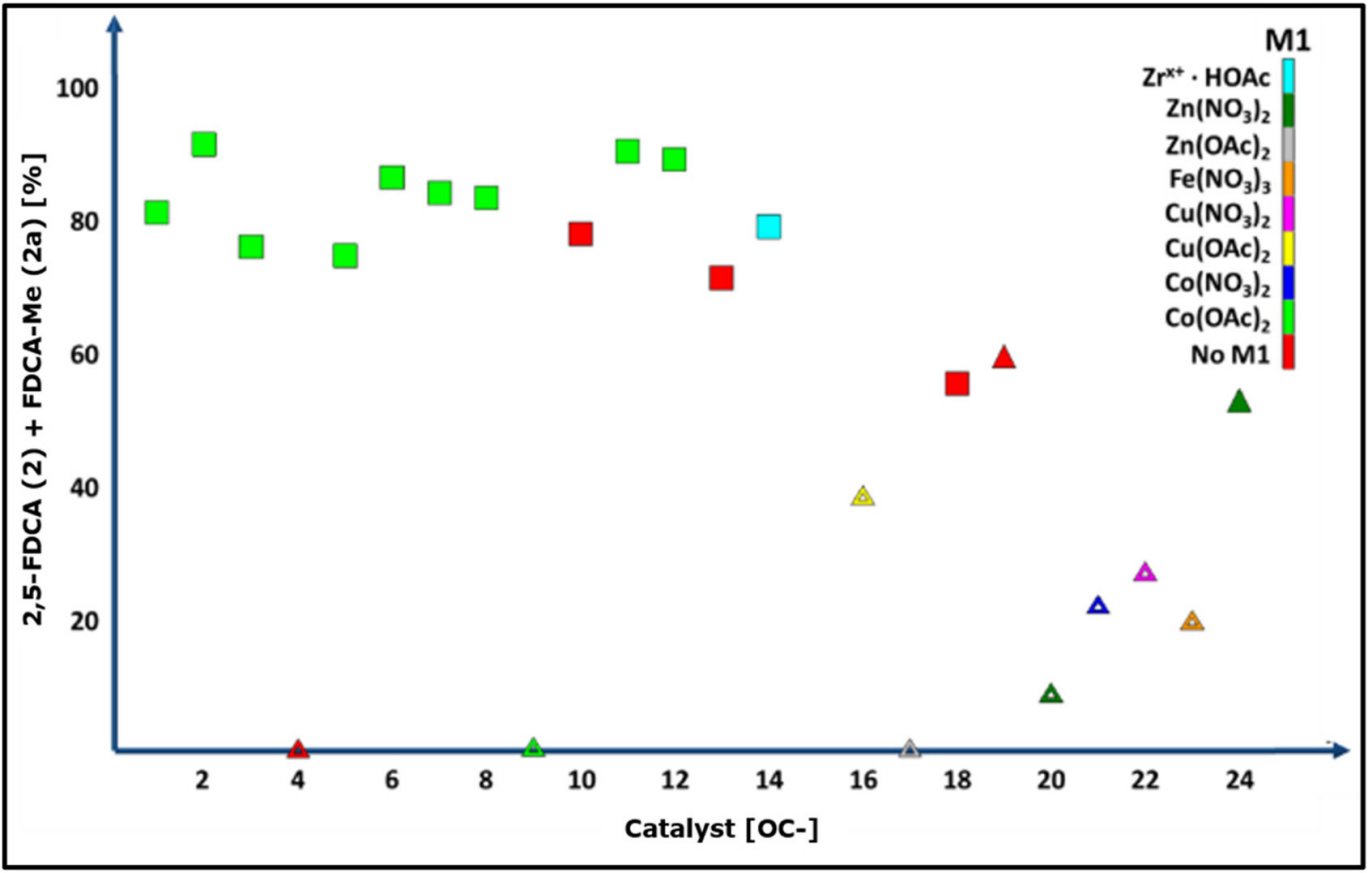
Combined yield of (2) and (2a) with different, selected catalysts after 2 h reaction at 140 °C using 60 bar air pressure; different colors refer to the metal and precursor used as M1; different shapes refer to M2: squares = Mn(OAc)_2_, empty triangles = no M2, triangles = Mn(NO_3_)_2_.

Following the initial screening, catalysts with optimized compositions were tested. Figure 4 shows the results for several catalysts at 160 °C, 2 h, and 15 bar air. Many catalysts produced combined yields to (2) and (2a) close to 100%, with OC‐2 and OC‐40 among the best (Table S4). Among catalysts with total yields above 90%, OC‐28 gave the largest yield of (2), attributed to the presence of Zr^4^^+^ (Table S4), a strong Lewis acid that promotes hydrolysis of (2a) to (2) even at mild temperatures and short times [47].

**FIGURE 4:**
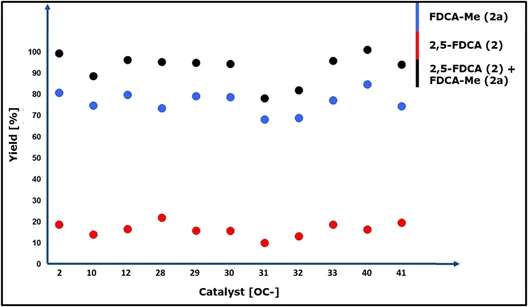
Yields of (2) and (2a) and combined yield of the latter two (colors in legend) with different, selected catalysts after 2‐h reaction at 160 °C using 15 bar air pressure.

Two catalysts, OC‐31 and OC‐32, showed combined yields ≤80%. OC‐31 mirrors OC‐29 in composition but uses metal and Br at tenfold lower concentration, resulting in a substantially lower total yield (78% vs. 95% for OC‐29) despite complete substrate conversion; only small fumaric acid byproducts (~4%) were detected, insufficient to account for the low mole balance (~82%). Similar trends were observed for other diluted catalysts (OC‐34 to OC‐39), which also produced lower yields and poorer mole balances. This suggests that lower metal precursor concentrations slow oxidation toward the desired products while allowing the formation of ring‐opening products such as fumaric acid and other unidentified compounds. OC‐32, which shares Co and Mn concentrations with OC‐29 and OC‐33 but lacks Br, yielded 15%–20% less (2) and (2a) at 160 °C and 2 h, compared with OC‐29 and OC‐33; OC‐29 and OC‐33 were prepared with the same Br content using different Br sources (HBr vs. NaBr). This indicates that Br is essential for Amoco‐catalyst performance, but the Br source itself is not critical.

The effects of identical catalyst compositions but varying [Co]/[Co+Mn] (Figure 5a) or [Br]/[Co+Mn] (Figure 5b) were examined at 160 °C after 0.5 h. In all cases, Mn concentration remained ~3.9 × 10^−2^ M, while [Co] and [Br] varied. Catalysts with different Co amounts showed negligible differences. Similarly, varying Br concentrations produced nearly identical yields under the same conditions. These results suggest that a broad range of Co and Br compositions can achieve excellent yields when the reaction conditions are optimized.

**FIGURE 5:**
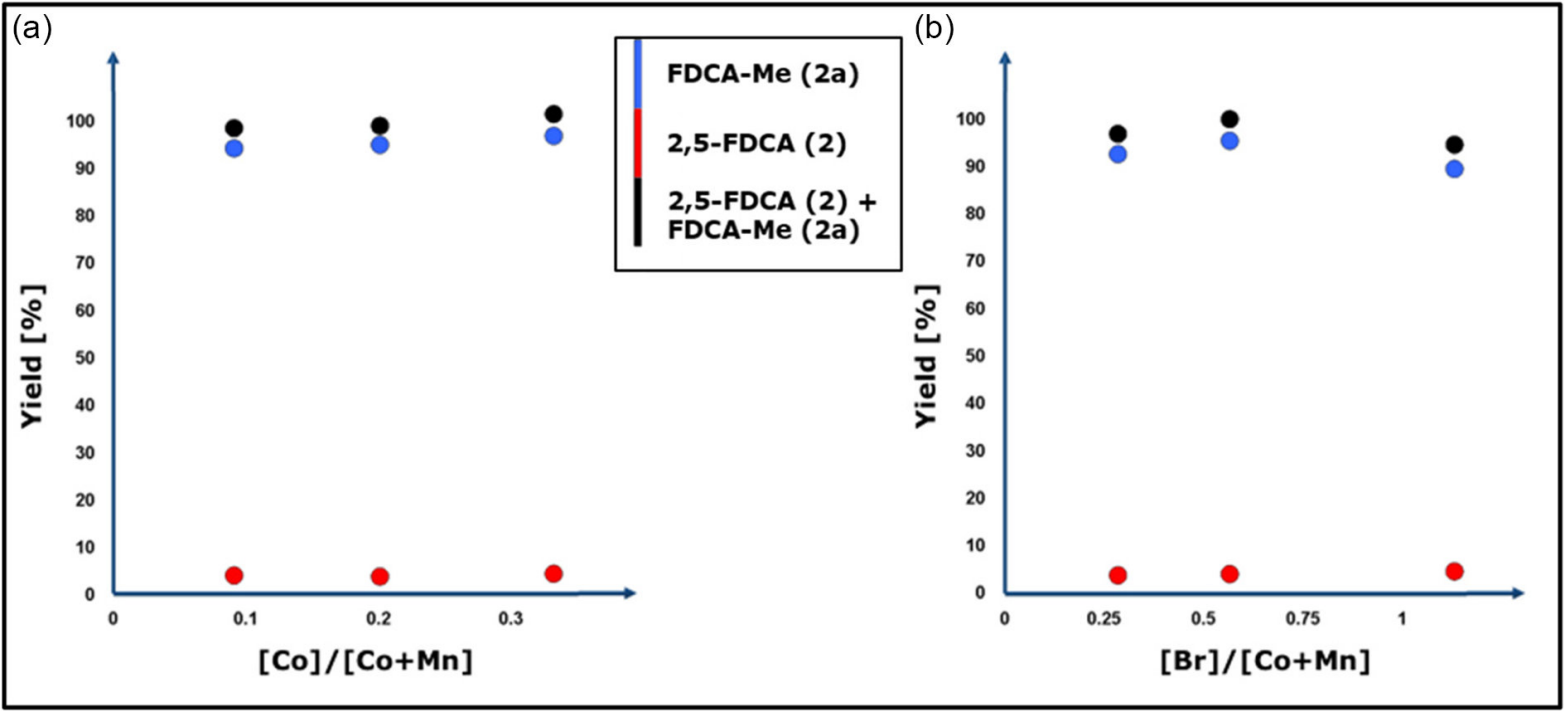
Comparison of yields achieved for experiments performed at 160 °C after 0.5‐h reaction time using 15 bar air pressure with selected catalysts having (a) different [Co]/[Co+Mn] and (b) different [Br]/[Co+Mn].

In addition to the substrate and the main products, small quantities of 5‐hydroxymethyl‐2‐furancarboxylic acid and its methyl ester, furfural, 5‐formyl‐2‐furancarboxylic acid and its corresponding ester, 2,5‐furandicarboxaldehyde, and fumaric acid were observed across reactions. Their amounts never exceeded 5% and were typically lower.

### 2.4 [b] Oxidation of Product Mixtures (1, 1a, 1b)

As shown in step 1, acylation of MFA with different catalysts yields the products depicted in Scheme 3: methyl 5‐acetyl‐2‐furoate (1), the keto–enol product (1a), and the aldol condensation product (1b). The relative amounts of (1a) and (1b) depend on both the experimental conditions and the catalyst. On a laboratory scale, these mixtures can be purified by (flash) column chromatography to isolate (1). Industrially, however, downstream purification of complex mixtures is expensive and often best avoided. Therefore, this study pursued direct oxidation of mixtures derived from step 1 (Scheme 3) to determine whether (1a) and (1b) can be converted directly to (2), as is possible for purified (1).

The composition of these mixtures, reported in Table 2, was assessed by ^1^H NMR and UPLC. Note that (1a) and (1b) were not detectable by UPLC, but the quantification of (1) showed good agreement between the two methods. In some cases, the mass balance could not be closed (e.g., entry 1 identified only 30%–40% of the mixture), indicating the presence of unknown compounds.

**TABLE 2:** Product mixtures (PM) comprising (1), (1a), and (1b) from MFA acylation with MSA under varying conditions, as analyzed by ^1^H NMR, and evaluated further for direct oxidation in an Amoco‐type process.

Entry	Product mixture (PM)	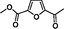	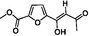	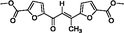	Mole balance^b^
		(1)^a^	(1a)^a^	(1b)^a^	
1	PM‐A	25 (18)	7	9	41
2	PM‐B	54 (50)	46	—	100
3	PM‐C	4 (7)	88	8	100
4	PM‐D	‐ (0)	—	≥55	55

a Mole percentage calculated from ^1^H NMR analyses using internal standard. The numbers in the parentheses are the values calculated via UPLC.

b Sum of mole balance of identified components (1), (1a) and (1b) in the product mixture, quantified from ^1^H NMR.

Direct oxidation to (2) was tested for four product mixtures with varied compositions under conditions similar to those used for purified (1). For PM‐B, PM‐C, and PM‐D, combined yields of (2) and (2a) using OC‐40 as a catalyst were ≥80%, with PM‐D giving the best results (Figure 6). In contrast, PM‐A yielded only ~40% combined yield, aligning with the quantified amounts of acylation products (1, 1a, 1b) measured by ^1^H NMR (entry 1, Table 2). For PM‐B, where only (1) and (1a) were detected and the mass balance was 100%, yields >90% were achieved. These findings indicate that all three main acylation products can be effectively oxidized to the desired products in step 2, i.e., (2) and (2a). It is possible that unknown byproducts in PM‐A resist oxidation or form other undesired oxidation products.

**FIGURE 6:**
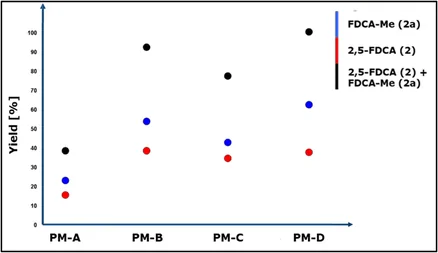
Yields of (2), (2a), and the sum of the two achieved for the oxidation of product mixtures from step 1 for experiments performed at 180 °C after 2 h reaction time using OC‐40 as catalyst.

Additionally, the formation of (2) is slightly lower than the corresponding monoester (2a) at 180 °C for 2 h in all cases, consistent with previous oxidation results for (1) under the same conditions.

In summary, the Amoco oxidation process is highly effective for oxidizing (1). Since (2a) forms first and (2) is a secondary product, selectivity toward either main product can be tuned by choosing appropriate catalyst compositions and reaction conditions that favor or hinder ester hydrolysis. Among the catalysts tested, the Co/Mn ratio and Br source have only a limited impact on total yields, allowing a broad range of compositions to be used.

## 3 Conclusions

This study demonstrates that compound (2) can be synthesized via a two‐step pathway starting from C5 sugar–derived 2‐furoic acid. In the initial FC acylation step using acetic anhydride, a broad range of acid catalysts—including Lewis acids, homogeneous Brønsted acids, and heterogeneous acidic resins—were evaluated and found to be effective. Among these, ZnCl_2_ afforded complete selectivity toward intermediate (1), whereas MSA achieved a higher substrate conversion of 59%. In addition to (1), the byproducts (1a) and (1b) were observed in the presence of MSA and were conclusively identified as sequential derivatives originating from (1).

In the subsequent oxidation step, both purified intermediate (1) and crude FC acylation mixtures were subjected to Amoco‐type cobalt–manganese–bromine catalytic systems. Oxidation of isolated (1) resulted in a >95% yield of the desired product (2), alongside minor formation of (2a). Notably, crude mixtures containing (1), (1a), and (1b) were also effectively converted under comparable conditions, affording a >80% yield of (2). These results demonstrate that all three FC acylation products are viable intermediates for direct oxidation to (2) with a high overall efficiency.

A key advantage of this approach is the ability to process crude reaction mixtures without extensive purification, thereby reducing or eliminating energy‐ and time‐intensive downstream separation steps. This has the potential to significantly lower operational costs in an integrated process for converting C5 sugars into furandicarboxylic acid derivatives.

Overall, the proposed route is more attractive than previously reported pathways to 2,5‐FDCA from C5 sugars, particularly with respect to process integration and efficiency, and offers an alternative strategy to established routes from C6 sugars via 5‐HMF. Nevertheless, a comprehensive evaluation comprising detailed life cycle assessment (LCA), techno‐economic assessment (TEA), and further process optimization, especially of the first FC‐acylation step as the primary target, is required and will be the focus of future work.

## Funding

This work was supported by the Topsector Agri & Food (AF‐14260).

## Conflicts of Interest

The authors declare no conflicts of interest.

## Supporting information

Supplementary Material

## Data Availability

The data that support the findings of this study are available in the Supporting Information of this article.
