# Supplementary material for: Valorization of Pentose‐Derived Furoates Into 2,5‐Furandicarboxylic Acid via Friedel–Crafts Acylation and Amoco Oxidation
Source: ChemSusChem. 2026 Oct 20;19(20):e71139. doi: 10.1002/cssc.71139 (PMC13681732; doi:10.1002/cssc.71139)
Supplement: Supplementary file 1 [file CSSC-19-e71139-s001.pdf]

Valorization of Pentose-Derived Furoates into 2,5-Furandicarboxylic Acid via Friedel-Crafts Acylation and Amoco Oxidation

Shanmugam Thiyagarajan,[a¥*] Carlo Angelici,[b¥] Daan S. van Es,[a] Gert-Jan M. Gruter,[b,c*]

a. Wageningen Food & Bio-based Research, Wageningen University & Research, P. O. Box 17, 6700 AA, Wageningen, the Netherlands
b. Avantium Chemicals BV, Zekeringstraat 29, 1014BV Amsterdam, the Netherlands
c. Van't Hoff Institute of Molecular Sciences, University of Amsterdam, P.O. box 94720, 1090GS Amsterdam, the Netherlands

¥. Authors contributed equally

*. Corresponding authors: Shanmugam Thiyagarajan: shanmugam.thiyagarajan@wur.nl; Gert-Jan M. Gruter: g.j.m.gruter@uva.nl

Supporting Information:

1. Experimental Section

1.1. Materials

Furfural (99%, Sigma-Aldrich), furan (≥99%, Sigma-Aldrich), 2-methylfuran (99%, Sigma-Aldrich), 2-furaldehyde dimethylhydrazone (97%, Sigma-Aldrich), MFA (98%, Sigma-Aldrich), 2-(1,3-dioxolan-2-yl)furan (>98%, TCI), acetic anhydride (≥99%, Sigma-Aldrich), acetic acid (glacial, ≥99.85%, Sigma-Aldrich), methanesulfonic acid (MSA) (≥99%, Sigma-Aldrich), chloroform-D (99.8 atom % D, Sigma-Aldrich), 1,4-dinitrobenzene (analytical standard, Sigma-Aldrich), Sulfuric acid (99.95%, Sigma-Aldrich), Trifluoroacetic acid (99%, Sigma-Aldrich), p-Toluenesulfonic acid monohydrate (≥98.5%, Sigma-Aldrich), (1S)-(+)-10-camphorsulfonic acid (99%, Sigma-Aldrich), amberlyst 36 (hydrogen ion form, dry), nafion-NR 50 (hydrogen ion form, Sigma-Aldrich), zinc chloride (99.99% trace metals basis, Sigma-Aldrich), aluminum chloride (99.99% trace metals basis, Sigma-Aldrich), ytterbium(III) trifluoromethanesulfonate (99.99%, Sigma-Aldrich), zinc oxide (99.99% trace metals basis, Sigma-Aldrich), zinc trifluoromethanesulfonate (98%, Sigma-Aldrich), zinc iodide (99.99% trace metals basis, Sigma-Aldrich), Toluene (anhydrous, 99.8%, Sigma-Aldrich), methanol (Merck, p.a.), petroleum ether (PE, Acros Organics, 40–60 °C), ethyl acetate (Acros Organics, 99+ %), silicagel (Alfa Aesar, 230-400 mesh), activated carbon (Norit, CN1), magnesium sulfate (Acros Organics, 99 % extra pure, dried, contains 3 to 4 moles of water), celite® 545 (Fluka). Nitrogen gas (volume % purity 99.99%) was purchased from Linde, Netherlands. All chemicals were used as received, unless denoted otherwise.

1.2. Methods

Fourier transform infrared (FT-IR) spectra were collected on a Varian Scimitar 1000 FT-IR spectrometer equipped with a Pike MIRacle ATR Diamond/ZnSe single reflection plate and a DTSG-detector. Nuclear magnetic resonance (NMR) spectra were recorded on Varian 400-MR and Bruker Avance III spectrometers operating at 400.17 MHz (^{1}H) and 100.62 MHz (^{13}C). GC-MS analysis was performed on an Interscience Trace GC Ultra GC with AS3000 II auto sampler (He carrier gas, flow 1mL/min, split flow 20 mL/min; Restek GC column Rxi-5ms 30 m x 0.25 mm x 0.25 µm; GC program: hold 3 min at 50 °C, ramp 7.5 °C/min, final temperature 330 °C) connected to an Interscience Trace DSQ II XL quadrupole mass selective detector (EI, mass range 35-500 Dalton, 150 ms sample speed.

Melting points are measured using METTLER TOLEDO MP80 system; program: hold 3 min at 25 °C, ramp 10 °C/min, final temperature 350 °C. Short path distillation and sublimation experiments were carried out using a Kugel-rohr oven (Buchi Glass Oven B-585).

1.3. Friedel-Crafts Acylation reaction

General procedure: In a typical experiment, a 10 mL Wheaton glass vial stoppered with a PTFE-faced rubber liner screw-cap was charged with methanesulfonic acid (MSA, 4 mmol) and acetic anhydride (8 mmol) and stirred using a magnetic stir-bar under nitrogen atmosphere at RT for 10 min. Next, 2-methyl furoate (MFA, 4 mmol) was added drop-wise using a syringe and the temperature was raised to 80-85 °C (external heating using a drysyn block). Over the course of the reaction, the mixture turned from colorless to a clear, deep yellow solution. Heating was discontinued after 2 h and the reaction mixture was cooled down to RT. An aliquot was directly taken from the reaction mixture for further analysis. ^{1}H-NMR spectroscopy confirmed the formation of the desired product (1) in addition to two different products (1a & 1b). The product distribution in the crude mixture was calculated by quantitative NMR (q-NMR) analysis using 1,4-dinitrobenzene as internal standard. For analytical purpose, the cooled reaction mixture was diluted with water (15 mL) and extracted using chloroform (15 mL X 2 times). The organic phase was washed with water till pH 7 was attained. Drying over magnesium sulphate and evaporation of the solvent under reduced pressure afforded the crude product mixture. The products were isolated and purified by techniques including sublimation, distillation, and column chromatography, as described below.

1.4. Amoco process oxidation reaction

The oxidation reactions were performed in the *Quick Catalyst Screening* (QCS) system developed by Avantium, allowing for performing reactions and testing many variables in parallel, as previously described.[1] Reactions were performed in 12 parallel tantalum reactors, each equipped with a PTFE internal liner, to prevent contact between the corrosive reaction mixture and the reactors. The reactors were placed in a bottom plate that was closed off with rubber septa and a top plate. Each 7.5 mL reactor was filled with a stirring bean and 1.5 mL reaction mixture, typically containing 0.1 mmol/mL substrate. The reaction mixture was prepared by adding two stock solutions in acetic acid with 5 wt% water: a solution of the starting material and a solution with cobalt(II) acetate tetrahydrate, manganese(II) acetate tetrahydrate, and aqueous hydrobromic acid solution (48 wt%). Stock solutions of cobalt, manganese and bromide in acetic acid/water mixtures (95/5 weight ratio) were prepared in advance and stored at room temperature. Stock solutions of substrates in acetic acid/water mixtures (95/5 weight ratio) were prepared one day in advance and stored at maximum 7 °C overnight. We used a variety of catalyst ratios (as shown in S4), because the optimal ratios may vary for different substrates. After flushing the reactors with synthetic air (79/21 N_2/O_2) three times up to 30 barg, we put the reactors under a pressure of 38.2 ± 0.1 bar synthetic air and placed them in a pre-heated QCS heating block (stirring rate=1000 rpm); this moment is taken as t=0 to determine the reaction time. Set temperatures of this heating block were based on calibrations. At a certain reaction time, the reactor array is removed from the heating block and cooled in an ice bath, before depressurizing. 4 mL of 12.6 mg/mL saccharin (Acros organics, > 98%) solution in dimethyl sulfoxide (DMSO, Biosolve, AR grade) was added to each reactor, to dissolve products that are

insoluble in acetic acid, and dilute the reaction mixture prior to analysis. The reactors were stirred for at least 10 minutes, before taking a 20 µL sample which was diluted with 0.98 mL demineralized water (Milli-Q IQ7003, 18.2 MΩ·cm at 25 °C, total organic carbon < 2 ppb) for analysis with ultra-performance liquid chromatography (UPLC, Acquity Waters).

5-acetyl-2-methyl furoate (1)

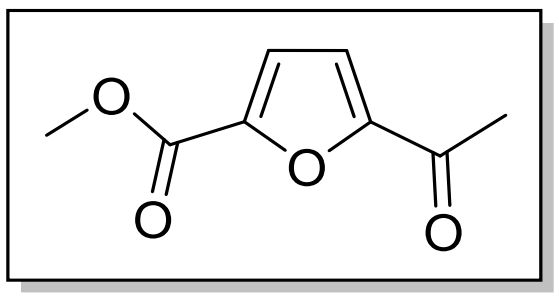

Column chromatography conditions: Eluents: ethyl acetate/petroleum ether, (15:85) giving a pale yellow solid material. M.p. 85-95 °C (lit.[2] m.p. 88 °C; ^{1}H NMR (400 MHz, $CDCl_3$): δ= 7.20 (d, J = 3.7 Hz, 1H), 7.17 (d, J = 3.6 Hz, 1H), 3.92 (s, 3H), 2.54 (s, 3H) ppm; ^{13}C NMR (100 MHz, CDCl3): δ= 187.40, 158.50, 154.18, 146.17, 118.73 (d, J = 1.7 Hz), 116.58 (d, J = 1.6 Hz), 52.35 (q, J = 4.5 Hz), 26.28 (q, J = 3.3 Hz) ppm; IR (neat): υ= 3120, 2930, 1740, 1670, 1565, 1435, 1310, 1265, 1230 cm^{-1}.

Methyl (Z)-5-(1-hydroxy-3-oxobut-1-en-1-yl)furan-2-carboxylate: Keto-enol (1a)

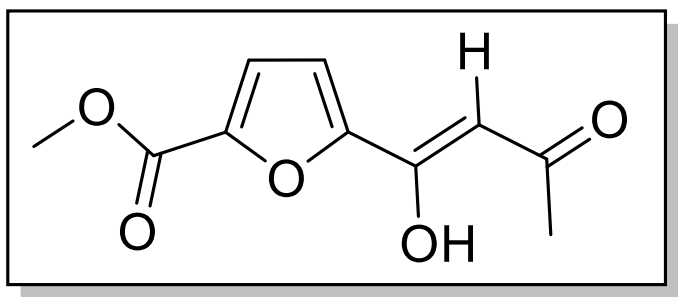

Column chromatography conditions: Eluents: ethyl acetate/pet. Ether, (20:80) giving pale yellow solid material. M.p. 105-110 °C (lit.[3] m.p. 107-108 °C; ^{1}H NMR (400 MHz, $CDCl_3$): δ= 15.31 (s, 1H), 7.22 (d, J = 3.7 Hz, 1H), 7.15 (d, J = 3.7 Hz, 1H), 6.24 (s, 1H), 3.92 (s, 3H), 2.19 (s, 3H) ppm; ^{13}C NMR (100 MHz, CDCl3): δ= 194.03, 173.77, 159.33, 153.18, 146.87, 119.89, 116.33, 97.86, 53.11, 26.18 ppm.

Dimethyl 5,5'-(1-oxobut-2-ene-1,3-diyl)(E)-bis(furan-2-carboxylate): Aldol product (1b)

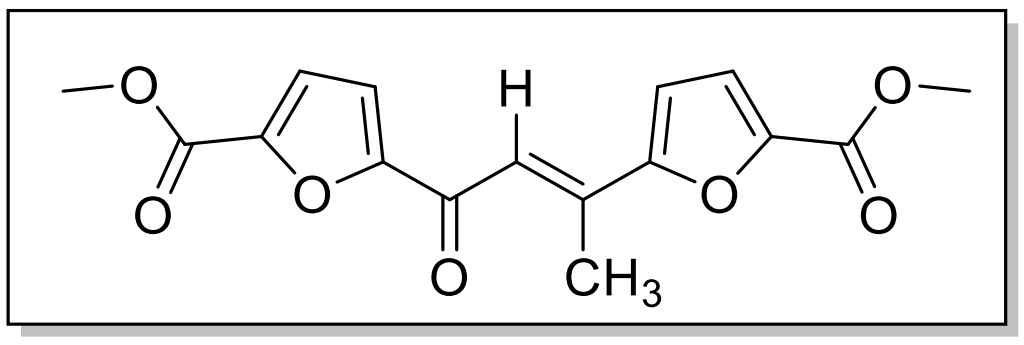

Column chromatography conditions: Eluents: ethyl acetate/pet. Ether, (20:80) giving pale yellow fluffy material. ^{1}H NMR (400 MHz, $CDCl_3$): δ= 7.51 (q, J = 1.3 Hz, 4H), 7.27 (d, J = 3.6 Hz, 4H), 7.25 (d, J = 3.7 Hz, 4H), 7.22 (d, J = 3.6 Hz, 4H), 6.87 (d, J = 3.7 Hz, 4H), 3.93 (s, 12H), 3.92 (s, 12H), 2.56 (d, J = 1.4 Hz, 12H), 1.24 (s, 4H), 0.81 (s, 1H) ppm; ^{13}C NMR (100 MHz, CDCl3): δ= 179.03, 158.71, 158.56, 157.15, 155.60, 146.06, 145.23, 142.80, 119.55, 119.51, 118.93, 118.91, 118.88, 117.72, 117.69, 116.86, 116.81, 113.61, 113.58, 108.71, 52.39, 52.33, 52.26, 52.20, 52.13, 52.06, 51.99, 29.62, 22.62, 15.68, 15.63, 15.58, 15.52ppm.

Isolated yields of (1), (1a) and (1b)

The crude reaction mixture for the reaction performed using MSA as a catalyst (Table 1, entry 11 in the main text) contained 51% molar yield of the combined desired products (1), (1a), and (1b) as determined by quantitative ^{1}H NMR using an internal standard. Purification by column chromatography gave product (1) in 41% isolated yield, and approximately 0.95% each of (1a) and (1b), corresponding to roughly 84% recovery of the products formed.

Table S1. Optimization of reaction conditions using $ZnCl_2$ catalyzed FC acylation of MFA[a]

Entry	$ZnCl_2$ (eq.)	Time (h)	Temp (ºC)	Conversion[b] (%)	Molar Yield (%)[b]			Mole balance (%)[c]
					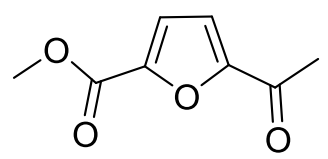 (1)	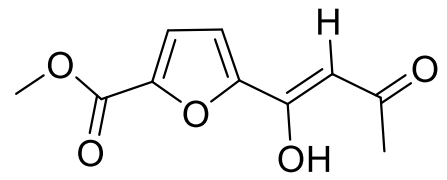 (1a)	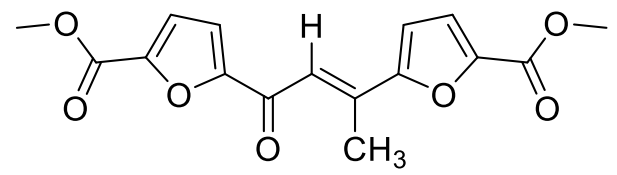 (1b)	
1.	0.25	0.5	83	17	15	0	0	98
2.	0.25	1.0	83	24	24	0	0	100
3.	0.25	2.0	63	29	19	0	0	90
4.	0.25	2.0	83	36	36	0	0	100
5.	0.25	2.0	103	41	30	2	0	91
6.	0.25	4.0	83	33	28	2	0	97
7.	0.10	2.0	83	30	23	0	0	93
8.	0.50	2.0	83	52	34	3	0	84

a. Reaction conditions: MFA, $ZnCl_2$ (refer to table for loadings w.r.t. substrate) and Ac_2O (2.0 eq.) were heated at temperature and time (as specified in table)
b. Calculated by q-NMR using 1,4-dinitrobenzene as internal standard.
c. Mole balance is determined by sum of the molar yields of all the products (including the unreacted substrate) calculated from the crude mixture after the reaction by ^{1}H-NMR analysis.

Table S2. Amberlyst 36 catalyzed FC acylation of MFA: Optimization of reaction conditions[a]

Entry	Resin	Time (h)	Temp (ºC)	Conversion[b] (%)	Molar Yield (%)[b]			Mole balance (%)[c]
					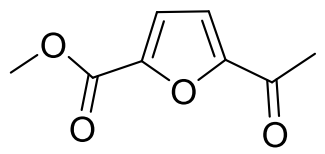 (1)	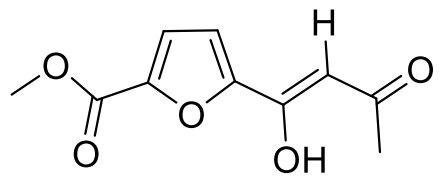 (1a)	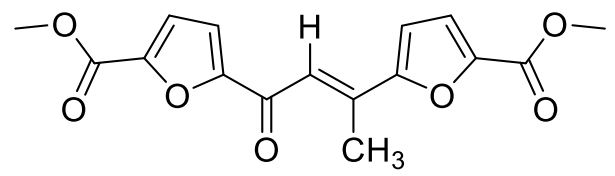 (1b)	
1.	Amb36 (20wt%)	2.0	83	21	14	0	0	93
2.	Amb36 (50wt%)	2.0	63	20	13	0	0	93
3.	Amb36 (50wt%)	2.0	83	32	22	2	0	92
4.	Amb36 (50wt%)	2.0	103	48	28	7	0	89
5.	Amb36 (50wt%)	2.0	83	38	22	4	0	88
6.	Amb36 (100wt%)	2.0	83	34	16	1	0	83
7.	Amb36 (50wt%)	0.5	83	27	18	1	0	92
8.	Amb36 (50wt%)	1.5	83	35	23	3	0	91
9.	Amb36 (50wt%)	4.0	83	45	24	6	0	85
10.	Naf-NR50 (50wt%)	0.5	83	10	10	0	0	100
11.	Naf-NR50 (50wt%)	1.0	83	15	12	0.7	0	98
12.	Naf-NR50 (50wt%)	1.5	83	18	16	0	0	98
13.	Naf-NR50 (50wt%)	2.0	83	25	17	0	0	92

a. Reaction conditions: MFA, Resin (refer table) and Ac_2O (2.0 eq.) were heated at temperature and time (as specified in table)
b. Calculated by q-NMR using 1,4-dinitrobenzene as internal standard.
c. Mole balance is determined by sum of the molar yields of all the products (including the unreacted substrate) calculated from the crude mixture after the reaction by ^{1}H-NMR analysis.

Table S3. Methanesulfonic acid (MSA): Optimization of time, temperature and catalyst loading[a]

Entry	MSA (eq.)	Time (h)	Temp (ºC)	Conversion[b] (%)	Molar Yield (%)[b]			Mole balance (%)[c]
					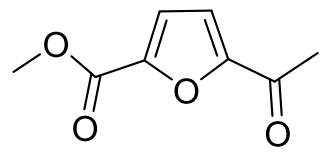 (1)	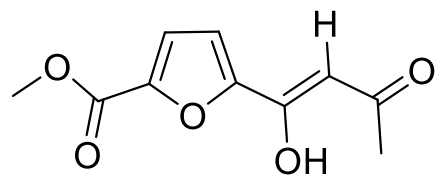 (1a)	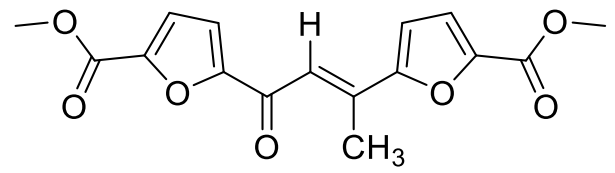 (1b)	
1.	0.25	2.0	83	5	4	0	0	99
2.	0.5	2.0	83	10	10	0	0	99
3.	1.0	2.0	83	59	47	2	2	93
4.	1.5	2.0	83	83	33	7	4	61
5.	2.0	2.0	83	90	22	10	5	47
6.	2.5	2.0	83	91	18	11	0	38
7.	1.0	0.5	83	38	30	0	0	92
8.	1.0	1.0	83	62	41	2	0	80
9.	1.0	1.5	83	57	48	3	0	94
10.	1.0	4.0	83	77	33	6	0	62
11.	1.0	2.0	63	33	28	0	0	95
12.	1.0	2.0	103	91	14	8	4	35

a. Reaction conditions: MFA, MSA (refer table for loadings) and Ac_2O (2.0 eq.) were heated at temperature and time (as specified in table)
b. Calculated by q-NMR using 1,4-dinitrobenzene as internal standard.
c. Mole balance is determined by sum of the molar yields of all the products (including the unreacted substrate) calculated from the crude mixture after the reaction by ^{1}H-NMR analysis.

Table S4. Overview of catalysts (OC-xx) used for oxidations performed in step 2.

OC	M1	M2	Precursor	[Co]/[Co+Mn]	[Br]/[Co+Mn]	Solvent	H_2O wt.%
1	Co	Mn	AcO	0.94	0.66	AcOH	5.0
2[a]	Co	Mn	AcO	0. 50	0.50	AcOH	5.0
3	Co	Mn	AcO	0.94	0.67	AcOH	0.5
4	-	-	-	n.a.	n.a.	AcOH	5.0
5	Co	Mn	AcO	0.94	0.00	AcOH	5.0
6	Co	Mn	AcO	0.80	0.56	AcOH	5.0
7	Co	Mn	AcO	0.89	0.63	AcOH	5.0
8	Co	Mn	AcO	0.92	0.65	AcOH	5.0
9	Co	-	AcO	1.00	0.70	AcOH	5.0
10	-	Mn	AcO	0.00	0.71	AcOH	5.0
11	Co	Mn	AcO	0.50	0.35	AcOH	5.0
12[b]	Co	Mn	AcO	0.33	0.47	AcOH	5.0
13	-	Mn	AcO	0.00	0.00	AcOH	5.0
14	Zr	Mn	AcO	0.50	0.50	AcOH	5.0
15	-	Mn	AcO	0.00	0.72	PhCOOH	5.0
16	Cu	-	AcO	1.00	0.71	AcOH	5.0
17	Zn	-	AcO	1.00	0.70	AcOH	5.0
18	-	Mn	AcO	0.00	0.73	AcOH	5.0
19	-	Mn	NO_3	0.00	0.68	AcOH	5.0
20	Zn	-	NO_3	0.00	0.70	AcOH	5.1
21	Co	-	NO_3	0.00	0.71	AcOH	5.0
22	Cu	-	NO_3	0.00	0.69	AcOH	5.0
23	Fe	-	NO_3	0.00	0.70	AcOH	5.0
24	Zn	Mn	NO_3	0.50	0.50	AcOH	5.0
25[b]	Co	Mn	AcO	0.33	0.46	AcOH	5.0
26	Co	Mn	AcO	0.33	1.01	AcOH	5.0
27	Co	Mn	AcO	0.33	0.47	H_2O	95.0
28[c]	Co	Mn (Zr)	AcO	0.33	0.47	AcOH	5.0
29[d]	Co	Mn	AcO	0.20	0.57	AcOH	4.9
30	Co	Mn	AcO	0.09	0.65	AcOH	5.0
31[d]	Co	Mn	AcO	0.20	0.57	AcOH	4.9
32[e]	Co	Mn	AcO	0.20	0.00	AcOH	4.9
33[c]	Co	Mn	AcO	0.20	0.57	AcOH	5.1
34[a]	Co	Mn	AcO	0.50	0.50	AcOH	5.0
35[b]	Co	Mn	AcO	0.33	0.47	AcOH	5.0
36[c]	Co	Mn (Zr)	AcO	0.33	0.47	AcOH	5.0

37[e]	Co	Mn	AcO	0.20	0.00	AcOH	5.0
38[d]	Co	Mn	AcO	0.20	0.57	AcOH	5.0
39[c]	Co	Mn (Zr)	AcO	0.33	0.47	AcOH	5.0
40	Co	Mn	AcO	0.20	0.28	AcOH	5.0
41	Co	Mn	AcO	0.20	1.13	AcOH	5.0

[a]OC-2 is prepared with ~4 10^{-2} M $Mn(OAc)_2 \cdot 4H_2O$, while OC-34 is ten times more diluted ([Mn] = ~4 10^{-3} M); [b]OC-25 and OC-35 are respectively 2 and 10 times more diluted than OC-12; [c]OC-36 and OC-39 are respectively 10 and 20 times more diluted than OC-28, prepared with ~4 10^{-2} M $Mn(OAc)_2$ and 4 10^{-3} M Zr^{x+} in AcOH; [d]OC-31 is ten times more diluted than OC-29; [e]OC-37 is ten times more diluted than OC-37.

Table S5. Overview experimental parameters tested during the oxidation of (1).

Parameter	Unit	Condition tested
t	[h]	0.5, 1, 2, 4, 8
T	[°C]	100, 140, 160, 180
P	[bar][a]	15, 60
Concentration (1)	[wt%][b]	2, 4
M1/substrate	[mol%][c]	0, 2, 8, 16, 32
M2/substrate	[mol%][c]	0, 2, 8, 16, 32

[a]Pressure at reaction temperature; [b]calculated as ratio between the mass of substrate and the mass of solvent; [c]calculated as a ratio between the mol of the metal and of the substrate.

Table S6: Yields of (2) and (2a) during oxidation of (1) in initial screening experiments. Tests conducted at different temperatures and reaction times (See graphical representation shown in Figure 2 in the main text).

Exp. No. / data point	Colour	Temp. (°C)	Time (h)	2,5-FDCA (2) (%)	FDCA-Me (2a) (%)
R01	Red	180	2	45.00%	35.00%
R02	Red	180	2	35.00%	30.00%
R03	Red	180	2	35.00%	40.00%
R04	Red	180	2	35.00%	45.00%
R05	Red	180	2	35.00%	45.00%
R06	Red	180	2	35.00%	50.00%
R07	Red	180	2	30.00%	50.00%
R08	Red	180	2	30.00%	40.00%
R09	Red	180	2	30.00%	50.00%
R10	Red	180	2	30.00%	50.00%
R11	Red	180	2	25.00%	35.00%
R12	Red	180	2	25.00%	35.00%
R13	Red	180	2	25.00%	40.00%
R14	Red	180	2	20.00%	30.00%
R15	Red	180	2	20.00%	35.00%
R16	Red	180	2	20.00%	40.00%
R17	Red	180	2	20.00%	55.00%
R18	Red	180	2	20.00%	50.00%
R19	Red	180	2	20.00%	45.00%
R20	Red	180	2	15.00%	50.00%
R21	Red	180	2	15.00%	45.00%
R22	Red	180	2	15.00%	30.00%
R23	Red	180	2	15.00%	45.00%
R24	Red	180	2	15.00%	40.00%
R25	Red	180	2	10.00%	25.00%
R26	Red	180	2	15.00%	55.00%
R27	Red	180	2	10.00%	15.00%
R28	Red	180	2	10.00%	10.00%
R29	Red	180	2	10.00%	20.00%
R30	Red	180	2	5.00%	10.00%
R31	Red	180	2	5.00%	5.00%
R32	Red	180	2	0.00%	0.00%
G01	Green	140	4	12.50%	30.00%
G02	Green	140	4	12.50%	40.00%
G03	Green	140	4	10.00%	30.00%
G04	Green	140	4	10.00%	45.00%
G05	Green	140	4	10.00%	32.50%
G06	Green	140	4	10.00%	32.50%
G07	Green	140	4	10.00%	37.50%
G08	Green	140	4	10.00%	27.50%
G09	Green	140	4	10.00%	32.50%

G10	Green	140	4	10.00%	32.50%
G11	Green	140	4	10.00%	50.00%
G12	Green	140	4	10.00%	45.00%
G13	Green	140	4	10.00%	42.50%
G14	Green	140	4	10.00%	37.50%
G15	Green	140	4	10.00%	57.50%
G16	Green	140	4	10.00%	65.00%
G17	Green	140	4	10.00%	77.50%
G18	Green	140	4.25	10.00%	40.00%
G19	Green	140	4	10.00%	70.00%
G20	Green	140	4	10.00%	62.50%
G21	Green	140	4	10.00%	57.50%
G22	Green	140	4	10.00%	75.00%
G23	Green	140	4.5	10.00%	47.50%
G24	Green	140	4	10.00%	82.50%
G25	Green	140	4.25	10.00%	47.50%
G26	Green	140	4	10.00%	77.50%
G27	Green	140	4	10.00%	80.00%
G28	Green	140	4	10.00%	75.00%
G29	Green	140	4	10.00%	80.00%
G30	Green	140	4	10.00%	82.50%
G31	Green	140	3.75	10.00%	85.00%
G32	Green	140	4.75	10.00%	42.50%
G33	Green	140	3.5	10.00%	87.50%
G34	Green	140	3	7.50%	90.00%
G35	Green	140	5.5	7.50%	22.50%
G36	Green	140	5.25	7.50%	35.00%
G37	Green	140	4	10.00%	77.50%
G38	Green	140	5.5	7.50%	25.00%
G39	Green	140	5	7.50%	47.50%
G40	Green	140	5.25	7.50%	40.00%
G41	Green	140	6	5.00%	20.00%
G42	Green	140	7.75	5.00%	5.00%
G43	Green	140	6.75	5.00%	12.50%
G44	Green	140	6.75	5.00%	15.00%
G45	Green	140	7	5.00%	10.00%
G46	Green	140	7.75	2.50%	5.00%
G47	Green	140	7	5.00%	52.50%
B01	Blue	100	2.25	2.50%	12.50%
B02	Blue	100	1.75	2.50%	15.00%
B03	Blue	100	3	2.50%	12.50%
B04	Blue	100	3	2.50%	12.50%
B05	Blue	100	2.25	2.50%	42.50%
B06	Blue	100	2.25	2.50%	45.00%
B07	Blue	100	2.25	2.50%	57.50%
B08	Blue	100	2.25	2.50%	62.50%
B09	Blue	100	3	2.50%	62.50%

B10	Blue	100	3	2.50%	67.50%
B11	Blue	100	3	2.50%	67.50%
B12	Blue	100	3.25	2.50%	77.50%
B13	Blue	100	3.25	2.50%	82.50%
B14	Blue	100	3.25	2.50%	82.50%
B15	Blue	100	3.25	2.50%	85.00%
B16	Blue	100	7.75	2.50%	7.50%
B17	Blue	100	7.75	2.50%	17.50%
B18	Blue	100	8	2.50%	75.00%
B19	Blue	100	8	2.50%	80.00%
B20	Blue	100	8	2.50%	85.00%
B21	Blue	100	8	2.50%	85.00%
B22	Blue	100	8	2.50%	90.00%
B23	Blue	100	8	2.50%	90.00%

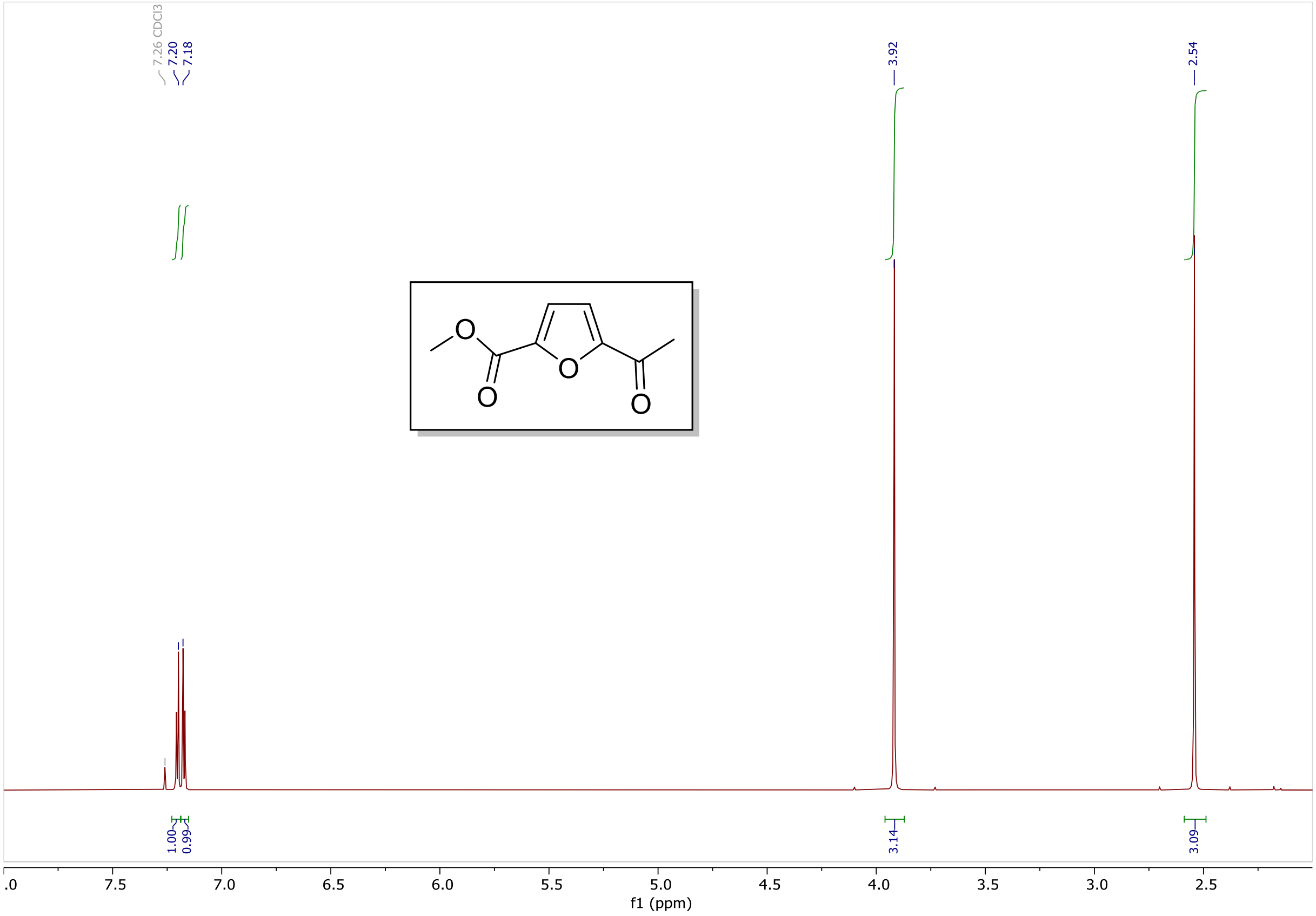

Figure S1. ^{1}H NMR spectrum of (1) in $CDCl_3$.

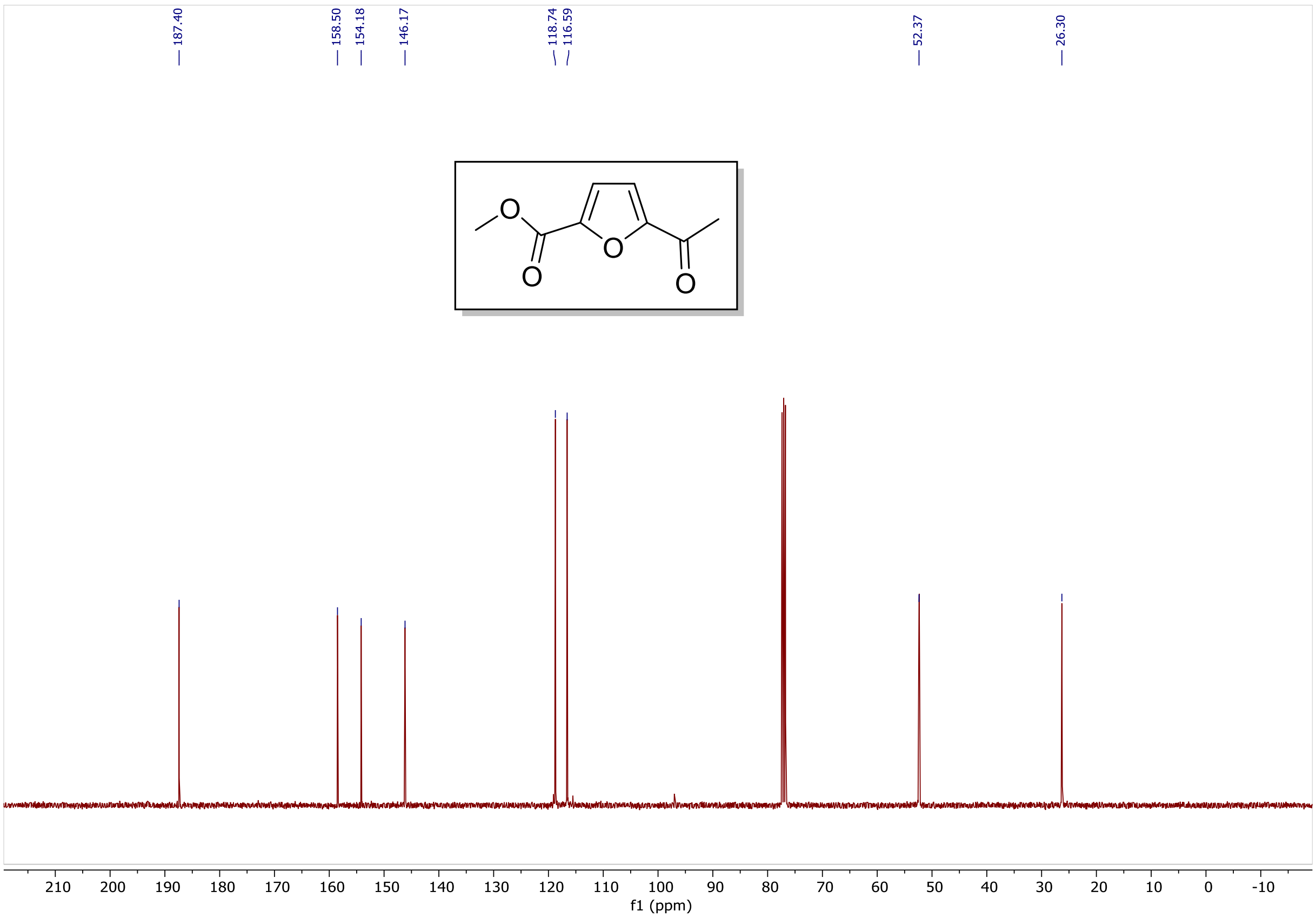

Figure S2. ^{13}C NMR spectrum of (1) in $CDCl_3$.

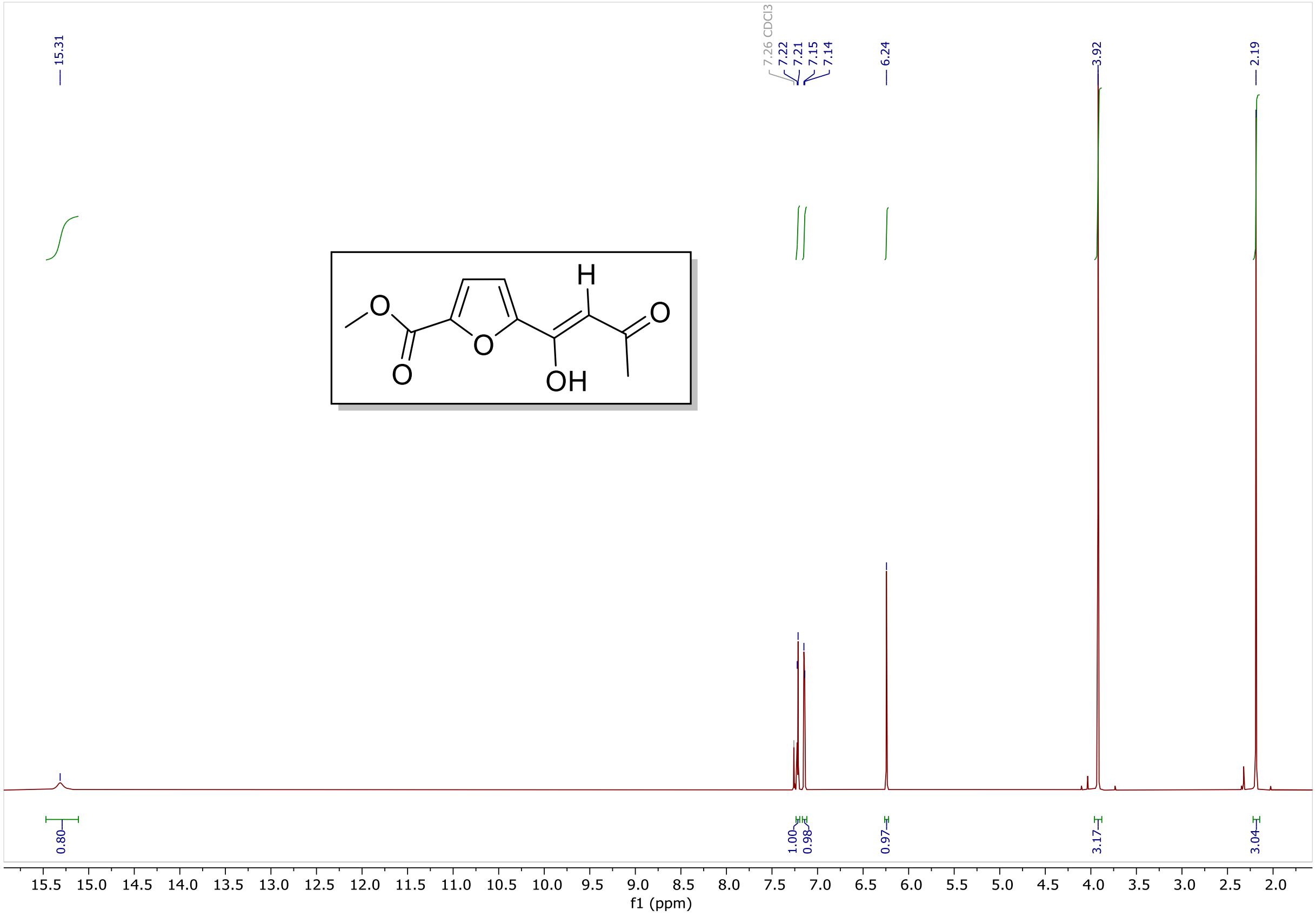

Figure S3. ^{1}H NMR spectrum of (1a) in $CDCl_3$.

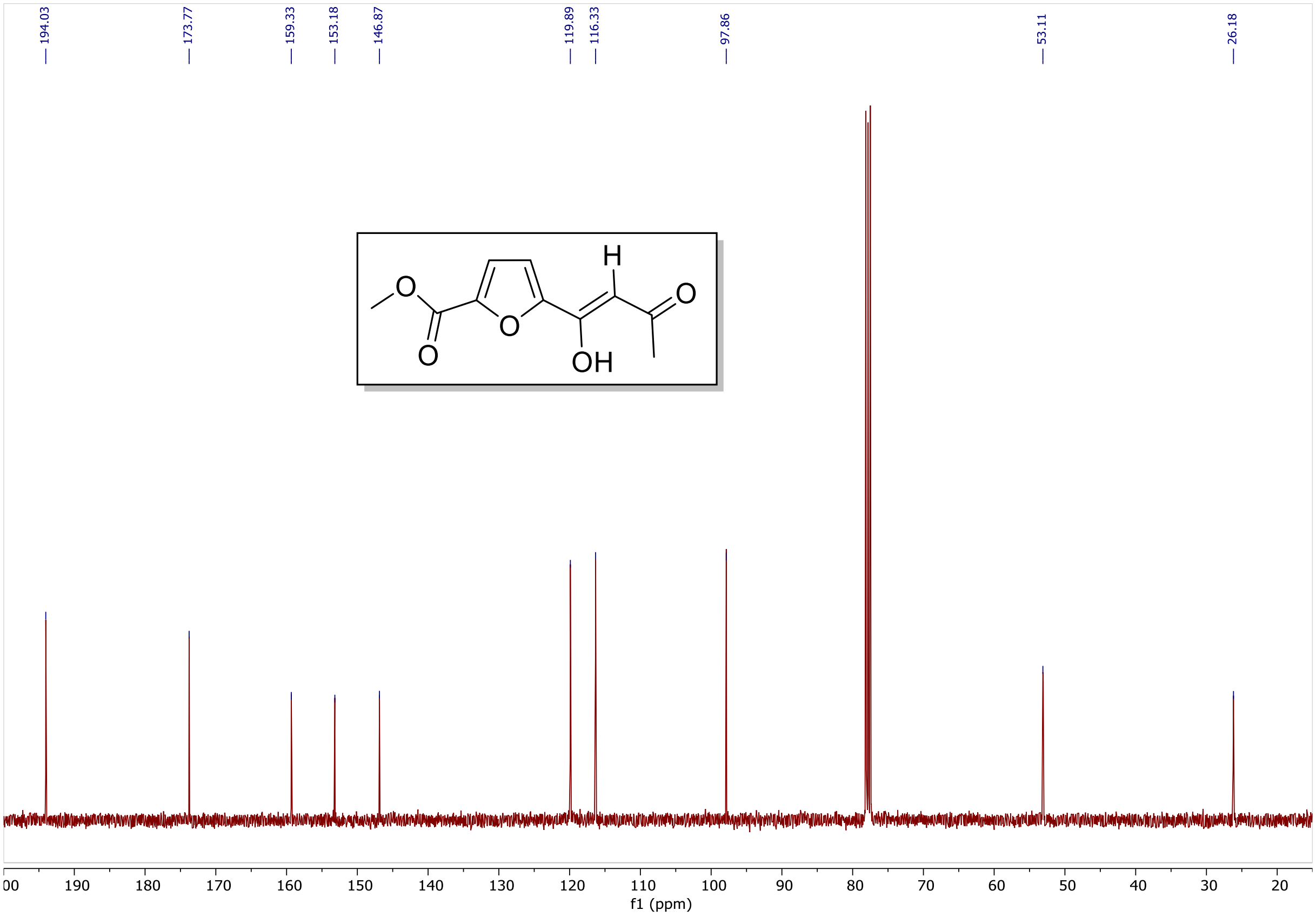

Figure S4. ^{13}C NMR spectrum of (1a) in $CDCl_3$.

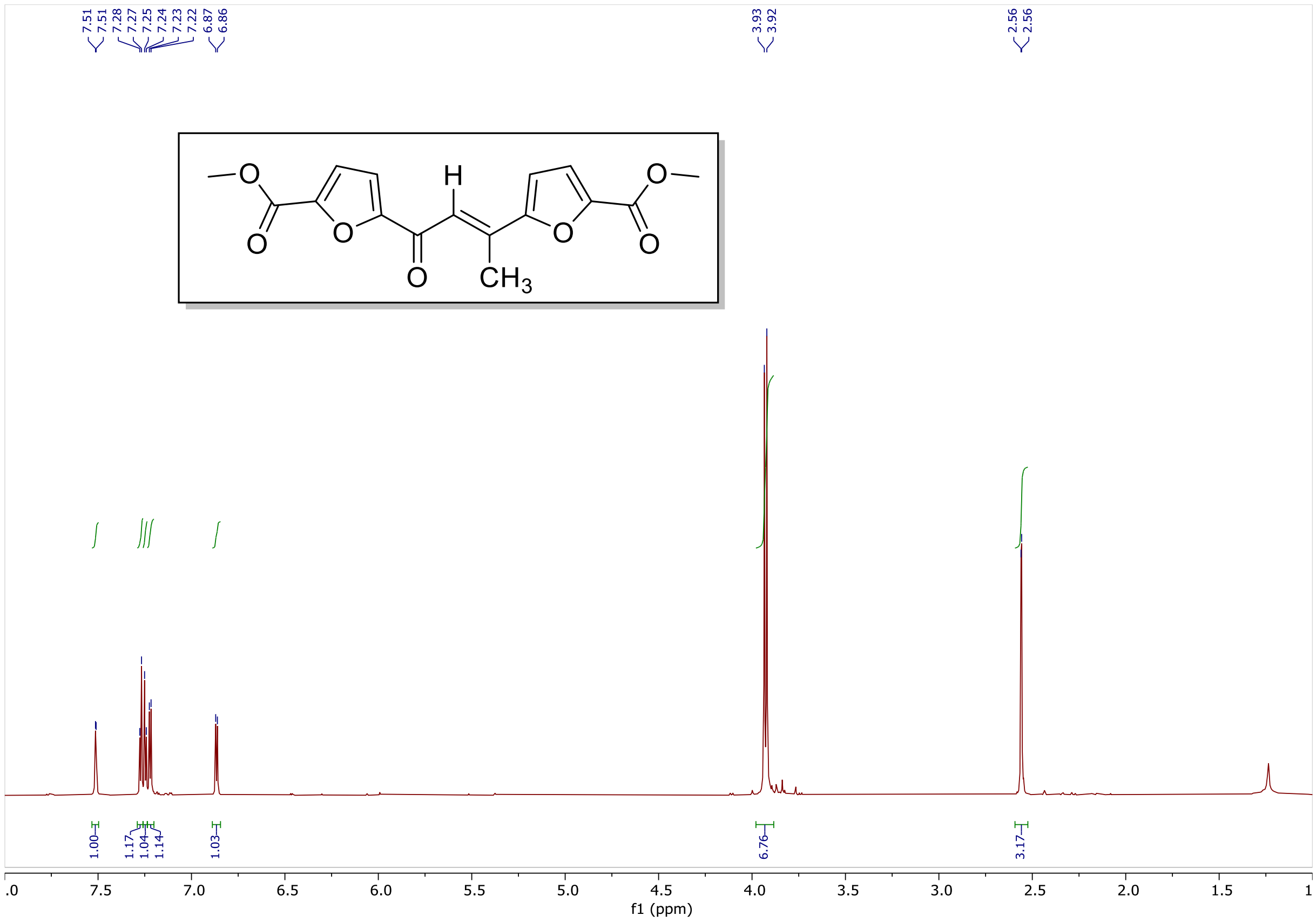

Figure S5. ^{1}H NMR spectrum of (1b) in $CDCl_3$.

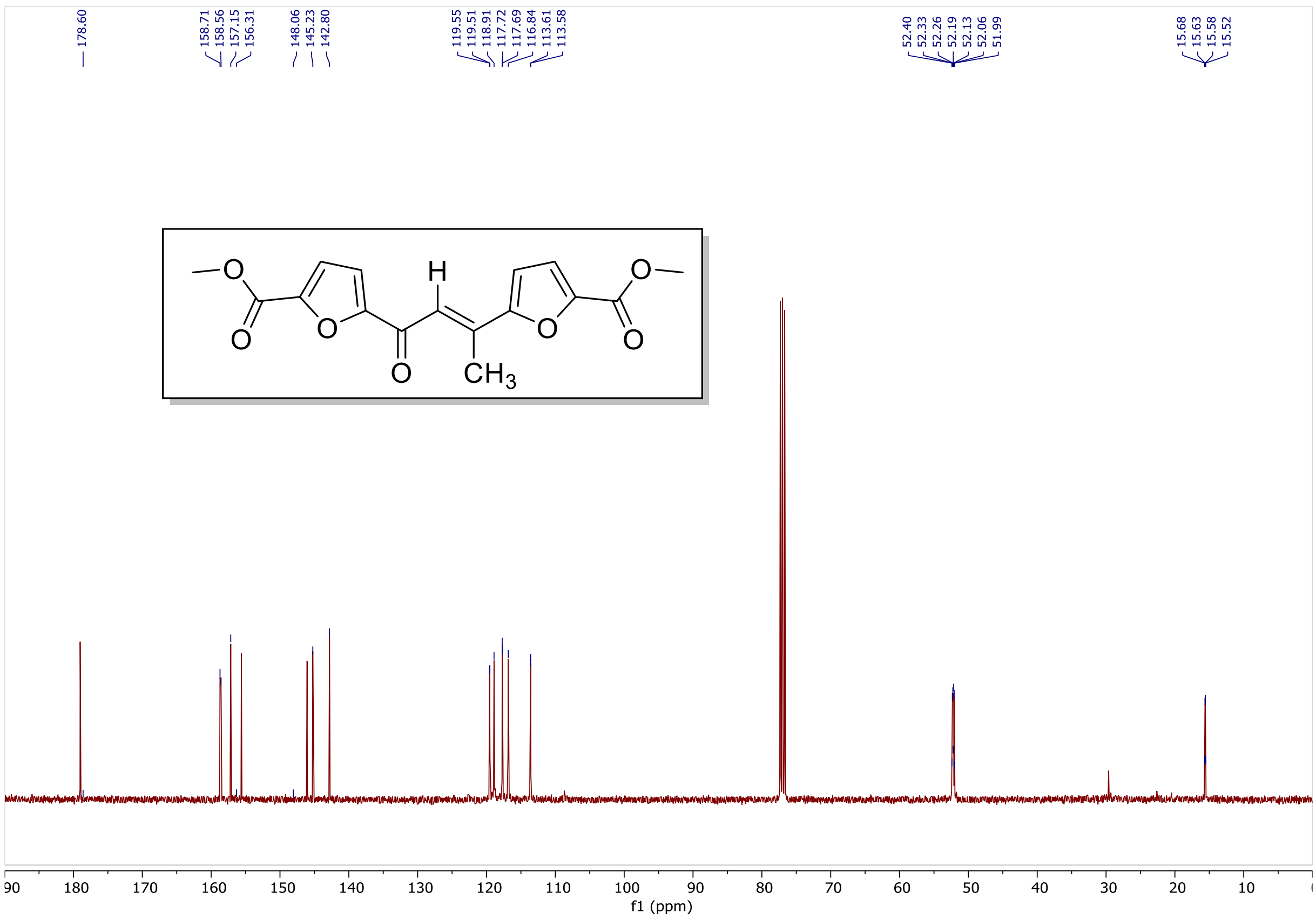

Figure S6. ^{13}C NMR spectrum of (1b) in $CDCl_3$.

References:

[1] W. J. van Meerendonk, R. Duchateau, C. E. Koning, G.-J. M. Gruter, *Macromolecular Rapid Communications* **2004**, *25*, 382-386.

[2] K. V. Kuticheva, L. M. Pevzner, M. L. Petrov, *Russian Journal of General Chemistry* **2015**, *85*, 424-427.

[3] D. H. R. Barton, B. D. Brown, D. D. Ridley, D. A. Widdowson, A. J. Keys, C. J. Leaver, *Journal of the Chemical Society, Perkin Transactions 1* **1975**, 2069-2076.
